# News sequences types of *Staphylococcus aureus* isolated from human pathologicals fluids in Burkina Faso

**DOI:** 10.1186/s13104-024-06805-9

**Published:** 2024-06-03

**Authors:** Roukiatou Traoré, Ganamé Abasse Ouédraogo, Abdoul Salam Ouédraogo, Aly Savadogo, Cheikna Zongo, Sylvain Godreuil

**Affiliations:** 1 Laboratoire de Biochimie et Immunologie Appliquées (LaBIA) au Burkina Faso, Université Joseph KI-ZERBO, 03 BP 7021 Ouagadougou 03, Burkina Faso; 2 Laboratoire de Bactériologie Hôpital Armaud de Villeneuve-CHU de Montpellier, 191 Avenue du Doyen Gaston Giraud, 34295 Montpellier Cedex 5, France; 3 Département de Bactériologie et de Virologie, Hôpital Universitaire Souro Sanou, Bobo-Dioulasso, Burkina Faso

**Keywords:** MRSA, Toxins, Sequence type, Antimicrobials, Burkina Faso

## Abstract

*Staphylococcus aureus* is a pathogen with high epidemic potential frequently involved in nosocomials and communities infections. The pathogenicity of *Staphylococcus aureus* is due to both its ability to resist antibiotics and to Produce toxins. This work aims at studying the resistance and Molecular Epidemiology of *Staphylococcus aureus.* Antibiotic susceptibility of the 70 strains isolates of Staphylococcus aureus was determined by agar diffusion while Multiplex PCR and MLST were used to search toxin-coding genes and MRSA typing, respectively. 14.28% of isolates were multidrug resistant. *Staphylococcus aureus* showed high susceptibility to aminoglycoside and Macrolides familly. *lukS-PV/lukF-PV* and *sea genes* were detected in 45% and 3% of *Staphylococcus aureus* respectively. Ten (10) sequence types including ST5710, ST2430, ST5289, ST5786, ST6942, ST6943, ST6944, ST6945, ST6946, ST6947 have been reported. The study showed a diversity of antibiotic resistance phenotypes and a great diversity of MRSA clones causing infections.

## Introduction

*Staphylococcus aureus* belongs human commensal flora and occurs worldwide as an important pathogen responsible for many nosocomial and community infections. It is found in both local and invasive infections with clinical outcomes ranging from simple asymptomatic colonization to life-threatening infections. *Staphylococcus aureus* is distinguished from other staphylococci by the expression of numerous toxins, adhesion molecules, enzymes and others virulence’s factors that allow it to invade its host and evade its immune defenses [23]. Among these toxins, Panton-Valentine Leukocidin (PVL) and Toxic Shock Toxin (TSST-1) are particularly worrying to the microbiologist and clinician. lukS-PV/lukF-PV gene has been associated in severe osteoarticular infections and in fatal necrotizing pneumonia [8]. The virulence of S. aureus is a delicate concern for health workers. Added to this is their multi-resistance to antibiotics which is a major challenge to public health worldwide. Indeed, *Staphylococcus aureus* is one of those bacteria’s that are developing increasing resistance to antibiotics [21]. MRSA has become endemic in many regions where it adds to the morbidity, mortality, and cost of care associated with hospital-acquired infections [2].

In Burkina Faso, few studies have been conducted on the detection of toxin genes in *Staphylococcus aureus* isolated from pathological fluids. Therefore, this work aims to establish the resistance profile and Molecular Epidemiology of *Staphylococcus aureus* strains. It is part of the contribution to the improvement of the management of bacterial infections.

## Materials and methods

### Specimen collection

Clinical isolates of *Staphylococcus aureus* were collected from laboratories. Each isolate was aliquoted into a cryotube containing strain preservation media. And forms were elaborated for the reporting of the data necessary for the study.

### Study framework

The strains were collected from 6 hospital centers in the two largest cities of Burkina Faso including Ouagaddugou and Bobo Dioulasso.

In Ouagadougou, the strains were collected at the Yalgado OUEDRAOGO university hospital, the National Public Health Laboratory, CMA Shiphra Laboratory and the Paul 6 Medical Center Laboratory, the Center Laboratory. In Bobo Dioulasso they were collected at the Sourou SANOU University Hospital Center and MURAZ Center.

### Inclusion criteria

The strains collected should have the identity Staphyococcus aureus which have been confirmed by the various bacteriology laboratories of the collection health centers. The strains should come from the pathology products of patients who consulted in the health centers where the collections took place. the pathology products included were Pus, blood, urine, vaginal samples.

### Exclusion criteria

Strains from patient pathology products transferred from another health center to our collection sites were not included in this study. In addition, strains of Staphylococcus aureus originating from sputum, cerebrospinal fluids and pathology products of pregnant women were excluded.

### Study period

The activities took place between January 2018 to December 2020.

### Microbiological study of *Staphylococcus aureus*

The samples collected were plated on solid medium (CNA agar) and examined after 24 h of incubation at 37 ℃, following the manufacturer’s recommendations. Confirmation of *Staphylococcus aureus* was performed using Matrix-Assisted Laser Desorption/Ionization Time-of-Flight (MALDI-TOF).

### Antibiotic susceptibility test

In all confirmed *S. aureus* isolates, antibiotic susceptibility was determined by agar diffusion method [3] with the following antibiotics: Penicillin G (1U), Ampicilline (2 µg), Amoxicillin (20 µg), Amoxiclav (20–10 µg), Ticarcilline (75 µg), Piperacillin (30 µg), Imipenem (10 µg), Cefalexin (30 µg), Cefamandazole (30 µg), Oxacillin (5 µg), Gentamycin (10 µg), Tobramycin (10 µg), Amikacin (30 µg), Tetracyclin (30 µg), Erythromycin (15 µg), Clindamycin (2 µg), Linezolide (10 µg), Trimethoprim/sulfamethoxazole (1.25–23.75 µg), Ofloxacin (5 µg), Rifaampycin (5 µg), Fusidic acid (10 µg), Fosfomycin 200 µg). The SIRscan automaton (2000) was used for digital reading and analyzed according to the EUCAST, 2019 guidelines. Susceptibility to methicillin was screened with the Cefoxitin disk diffusion method.

### Extraction of DNA by lyse method and protéinase K

Lysis of the test isolates was performed on Muller-Hinton medium. A homogeneous bacterial suspension with an optical density of approximately 1 MacFarland was realized and then centrifuged at 10000 rpm for 10 min. To the obtained pellet, 111 μL of lysis mix (lysostaphin/lysosyme) was added then vortexed. After incubation for 1 h at 37 °C in water bath, 100 μL of proteinase K was added then vortexed and reincubated for 1 h at 50 °C; then 10 min at 100 °C in water bath. To perform heat shock, the eppendorf was placed in the cold block at − 20 °C/10 min, then centrifuged at 10000 rpm for 10 min. The supernatant containing DNA was collected and stored at − 20 °C for the different PCRs [26].

### Detection of toxin genes (*lukS-PV/lukF-PV, sea* and* tst*-1) *by multiplex* PCR

Multiplex PCR was carried out as described previously [10] with modifications. An aliquot of 5 µL of DNA suspension was added to 35 µL PCR mixture consisting of 0.6 µL Hostart Taq Polymérase, 12.5 µL sterile distilled water, 4 µL PCR Buffer 10X, 1.6 µL MgCl2, 4 µL dNTP 1.25 Mm, 1.5 µL of 5 pmol each *sea*, *lukS-PV/lukF-PV* and *tst*, primers pro-duced by Sigma-Aldrich, Germany (Table 1). Multiplex Polymerase Chain Reaction assays were carried out with a negative control containing all of the reagents without DNA template. DNA amplification was carried out using thermocycler with the following thermal cycling profile: Initial denaturation at 94 °C for 15 min, denaturation 94 °C for 30 s, annealing 55 °C for 30 s, extension 72 °C for 1 min and final extension 72 °C for 7 min with a pro-grammable period of 25 cycles. After PCR amplification, 5 µL of PCR product was resolved by agarose gel electrophoresis.

**Table 1:** The sequence of primers used in this study

Amorces	Sequence	Size (pb)	Reference
*Nuc* gene			
*nuc1*	5′-GCGATTGATGGTGATACGGTT-3′	280	[5]
*nuc 2*	5′-AGCCAAGCCTTGACGAACTAAAGC-3′		
*lukS-PV/lukF-PV* gene			
*pvl-1*	5′-ATCATTAGGTAAAATGTCTGGACATGATCCA-3′	433	[14]
*pvl-2*	5′-GCATCAASTGTATTGGATAGCAAAAGC-3′		
*Tst* gene			
*tst-1*	5′-TTCACTATTTGTAAAAGTGTCAGACCCACT-3′	180	[14]
*tst-2*	5′-TACTAATGAATTTTTTTATCGTAAGCCCTT-3′		
*Sea* gene			
*sea*-1	5′-GAAAAAAGTCTGAATTGCAGGGAACA-3′	560	[14]
*sea*-2	5′-CAAATAAATCGTAATTAACCGAAGGTTC-3′		

### Molecular typing in *Staphylococcus aureus* isolates

*Staphylococcus aureus* isolates were typed by multilocus sequence typing (MLST). MLST was performed by amplifying and sequencing the amplicons of 7 housekeeping genes as previously described [9].

## Results

### *Staphylococcus aureus* antimicrobial susceptibility testing

A total of 70 strains of *Staphylococcus aureus* isolated from pus (67%), from urine (14%), from vaginal swabs (10%) and from blood cultures (9%).

### Resistance of *Staphylococcus aureus* to *Beta*-lactam

Most (95%) of the strains showed resistance to at least one β-lactam antibiotic. The antibiotics to which the strains were most susceptible (81–87%) were Amoxicillin-clavulanicacid, Cefalexin, Cefamadazole, Imipenem and Cefoxtazime. The highest resistance (83–94%) was observed with Oxacillin, Ampicillin, Amoxicillin, Ticarcillin, Penicillin G and Piperacillin (Table 2). Among *Staphylococcus aureus* isolates, 17% methicillin-resistant *Staphylococcus aureus* strains were identified and 14% were multidrug resistant.

**Table 2:** Evaluation of the efficacy of different antibiotic families on *Staphylococcus aureus*

Familly	Antibiotic	Sensitivity N (%)	Resistance N (%)
β-lactam	Penicillin G	4 (6)	66 (94)
	Oxacillin	58 (83)	12 (17)
	Amoxicillin	4 (6)	66 (94)
	Ticarcillin	4 (6)	66 (94)
	Piperacillin	4 (6)	66 (94)
	Imipenem	58 (83)	12 (17)
	Cefalexcin	58 (83)	12 (17)
	Cefamandazole	58 (83)	12 (17)
	Cefoxtaxin	58 (83)	12 (17)
	Ampicillin	5 (7)	65 (93)
	Cefoxcitine	58 (83)	12 (17)
	AMC	57 (81)	13 (18.57)
Aminoglycoside	Kanamycine	62 (89)	7 (11)
	Amikacin	61 (87)	9(13)
	Gentamycin	69 (99)	1 (1)
	Tobramycine	68(97)	2 (3)
Macrolides	Erythromycin	57 (81)	13 (19)
Lincosamide	Clindamycin	70 (100)	0 (0.00)
Sulfamide	SXT	70 (100)	0 (0.00)
Quinolone	Ofloxacine	54 (77)	6 (23)
Others	Rifampycin	68 (97)	2 (3)
	Fusidic acid	68 (97)	2 (3)
	Fosfomycin	69 (99)	1 (1)

*AMC* amoxicillin- clavulanicacid, *SXT* trimethoprim/sulfamethoxazole

The high rates of susceptibility of strains to Aminosides, Lincosamides and Macrolides show the good activity of these molecules on *Staphylococcus aureus* circulating in health centers in Burkina Faso. For this purpose, the activity rates of the aminoglycoside molecules on the strains were 99% (Gentamycin), 98% (tobramycin), and 89% (kanamycin). However, one (1%) strain was KTG phenotype, one (1%) was KT phenotype, and six (9%) were K phenotype. Regarding the macrolide and lincosamides family, all strains were sensitive to clindamycin and 18.57% of strains were resistant to erythromycin.

### Resistance of *Staphylococcus aureus* to other antibiotic families

The susceptibility of *Staphylococcus aureus* to other antibiotic’s families showed strains sensitive to trimethoprim/sulfamethoxazole (100%), fosfomycin (99%), fusidic acid (97%), rifampycin (97%), ofloxacin (77%) and linezolid (100%).

### Presence of toxin encoding genes (*lukS-PV/lukF-PV, tst, sea*)

The *lukS-PV/lukF-PV* gene was detected from 45% of the strains analyzed with a much higher rate of lukS-PV/lukF-PV -positive MSSA isolates (58%) than lukS-PV/lukF-PV-positive MRSA (25%). The presence of the *lukS-PV/lukF-PV* gene (57%) was associated with suppurative skin and soft tissue infections including surgical wound infections and limb fracture wounds (p = 0.01285).

The sea gene was detected from 3% of isolates and the tst gene was absent in all isolates tested. The *sea* gene was detected from a strain responsible for vaginal infection (Table 3).

**Table 3:** Toxin-coding gene at each infection site

Infections sites	Presence of the *lukS-PV/lukF-PV-*gene N (%)	Presence of the *sea*-gene N (%)
Ears	1 (7)	–
Arrin	2 (14)	–
Bacteremia	2 (14)	–
Vaginal infection	1 (7)	1 (100.00)
Suppurative skin and soft tissue infection	8 (57)	–

### Analysis of strains by multi locus sequence type (MLST)

**Fig. 1:**
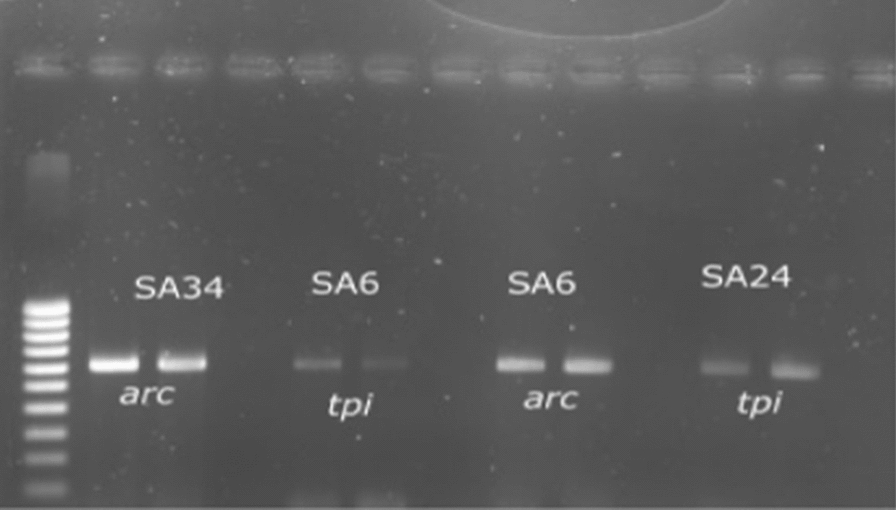
Migration gel of the arc gene of SA34 and SA6 strains and the tpi gene

Molecular typing by MLST was performed on twelve MRSA strains. The PCR amplification of the seven (7) housekeeping genes was verified by gel electrophoresis (Fig. 1).

MLST analysis of the strains clustered the twelve (12) MRSA isolates into ten (10) sequences types (ST5710, ST2430, ST5289, ST5786, ST6942, ST6943, ST6944, ST6945, ST6946, ST6947) whose allelic profiles are summarized in Table 4. Isolates SA32 and SA7 were assigned to ST5710, and isolate SA31 was assigned to ST2430. Isolate SA6 was assigned to ST5786. Isolate SA9 was assigned to ST5289. Isolates SA2, SA4, SA5, SA34, SA12, SA21, and SA33 were assigned to new STs ST6942, ST6943, ST6945, ST6946, ST6947, and ST6944 respectively and filed in *Staphylococcus aureus* typing database.

**Table 4:** Allelic profiles of typed MRSA strains

Strains	Allelic profiles							ST
	*Arc*	*Aro*	*glp*	*gmk*	*Pta*	*tpi*	*Yqi*	
SA21	22	853	14	23	707	770	747	ST6944
SA31	6	5	14	98	7	14	931	ST2430
SA32	22	853	14	23	707	4	31	ST5710
SA2	3	971	99	84	4	711	879	ST6942
SA4	803	964	818	84	559	745	929	ST6943
SA33	22	782	14	23	559	770	747	ST6944
SA5	79	716	671	23	707	770	698	ST6945
SA7	84	971	14	23	707	774	929	ST5710
SA34	6	5	671	98	755	14	99	ST6946
SA6	46	75	49	44	13	68	788	ST5786
SA9	22	958	14	400	679	770	700	ST5289
SA12	231	716	14	98	707	770	700	ST6947

*ST* Sequence Type

In summary, clones ST2430, ST6946, and ST5786, isolated from hospital settings, were found to be lukS-PV/lukF-PV-positive clones and less resistant to antibiotics. Clones ST6943, ST5710, and ST5289, isolated in community settings, are lukS-PV/lukF-PV-negative and multiply resistant to antibiotics (Table 5). Clones ST6942 and ST6945 are lukS-PV/lukF-PV-negative, multidrug-resistant hospital clones.

**Table 5:** Summary of molecular characteristics and resistance patterns of MRSA strains

Strains	Origin	Infectious site	Sample	Gender	Age (yrs)	*Lpv*	*Tst*	*sea*	ST	Non-β-lactam resistance
SA21	MRSA-H	Surgical wound	Pus	M	42	–	–	–	ST6944	Tet
SA31	MRSA-H	Surgical wound	Pus	F	20	+	–	–	ST2430	Tet
SA32	MRSA-H	Ulcerous lesion	Pus	F	20	–	–	–	ST5710	Tet
SA2	MRSA-H	Limb fracture	Pus	M	27	–	–	–	ST6942	Tet + K + Tob + Gen + Ak + Ery + Ofl
SA4	MRSA-C	Vaginal infection	PV	F	18	–	–	–	ST6943	Tet + Ofl + Ra
SA33	MRSA-H	Surgical wound	Pus	F	34	–	–	–	ST6944	Tet
SA5	MRSA-H	Blood cultures	Sang	M	21	–	–	–	ST6945	Tet + Ofl + Ra
SA7	MRSA-C	Ulcerous lesion	Pus	F	2	–	–	–	ST5710	Ak + Ery
SA34	MRSA-H	Surgical wound	Pus	M	25	+	–	–	ST6946	Tet
SA6	MRSA-H	Ulcerous lesion	Pus	M	83	+	–	–	ST5786	–
SA9	MRSA-C	Ulcerous lesion	Pus	M	30	–	–	–	ST5289	Tet + Da
SA12	MRSA-H	Limb fracture	Pus	F	17	–	–	–	ST6947	Tet + Ofl

## Discussion

The *Staphylococcus aureus* isolates from various clinical samples indicative that it iswidely distributed. The results of the resistance to antibiotics obtained show a significant proportion of resistant strains. Most 95% of the strains showed resistance to at least one β-lactam antibiotic. The persistent resistance of *Staphylococcus aureus* strains to the antibiotics of β-lactam could be due to the common use of these antibiotics by patients often without medical prescription. The strains were most susceptible with rates 81–86% to Amoxicillin-clavulanic acid, Cefalexin, Cefamadazole, Imipenem and Cefoxtazime. This shows that these antibiotics of the β-lactam family can remain a viable alternative for the treatment of *Staphylococcus aureus* infections. The highest resistance rates variables 83–94%, observed with Ampicillin, Amoxicillin, Ticarcillin, Penicillin G and Piperacillin. These resistances could be explained by intrinsic or acquired resistances. The rate to 36% of resistance to amoxicillin clavulanic acid was showed also by Koinam et al. [18] patients fluids and Kengne et al. [17] were reported 89.6% of *Staphylococcus aureus* isolates in pus resistance to penicillin G. Additional, *Staphylococcus aureus* isolated showed high susceptibility to aminoglycoside antibiotics. Indeed, one (1%) strain was KTG phenotype, one (1%) KT phenotype, and six (9%) were K phenotype. As this study, Kengne et al. [17] in Cameroon, Koinam et al. [18] in Burkina and Sina et al. [27] in Benin were reported *Staphylococcus aureus* isolates resisted to aminosides antibiotics. Three phenotypes were reported by Kengne et al. [17] but at different rates of resistance (10.4, 4.2, and 12.5% for the KTG, KT, and K phenotypes respectively). The presence of the K, KT and KTG phenotypes would be due to acquired resistance which is ensured by the production of inactivating enzymes in the strains [11].

Macrolides and licosamides have been reported to be successful antistaphylococcal agents. This study revealed a 100% susceptibility rate for clindamycin and only 18% of strains were resistant to erythromycin. Kahsay et al. [16] reported 97.2% resistance rate for erythromycin and only 5.6% of the strains were susceptible to clindamycin. Tsige et al. [29] found resistance rates of 4.3 and 26.1% for clindamycin and erythomycin respectively.

The susceptibility of *Staphylococcus aureus* isolates to trimethoprim/sulfamethoxazole, fosfomycin, fusidic acid, rifampycin, ofloxacin and Linezolid were to 100, 99, 97, 97, 77, and 100% respectively. Maharjan et al. [20] and Gitau et al. [12] were reported highly sensitive rates 99 and 97.3% to Linezolid in *Staphylococcus aureus* isolates respectively. The sensitive was reported for trimethoprim/sulfamethoxazole in Kenya [12], in Benin [27] and the United States [6] at 56.9, 35.6 and 98% respectively. Similar results were reported by Sina et al. [27] for susceptibility to fosfomycin (81.5%), fusidic acid (87.5%) and rifampycin (91.5%). However, other reports showed high resistance to fosfomycin (70%) and fusidic acid (64%) [13]. No resistance to ofloxacin in Cameroon [4].

The *lukS-PV/lukF-PV* genes were present 45% of isolates in the present study. Similar results were reported by Ouedraogo et al. [24] (44.9%) in Burkina. The role of this toxin may have an impact on the virulence of these strains. The present study identified a much higher rate of lukS-PV/lukF-PV-positive isolates compared to other countries. According to some studies, the prevalence of lukS-PV/lukF-PV-positive isolates is 2.85% in Benin [28], 20% in Malaysia [1], 19% in Iran [19]. Higher rates (51.5–61.4%) have been reported in Lithuania and Gambia [7, 25].

The presence of the *lukS-PV/lukF-PV-*gene (57%) was associated with suppurative skin and soft tissue infections p = 0.01285. In Benin PVL toxin was particularly prevalent in strains isolated from furuncles (89.5%) and pymyositis patients (89.2%) [27].

The *sea* gene was detected from 3% of isolates to responsible for vaginal infection. This rate is low but should not be neglected. Ouedraogo et al. [24] in his study on *S. aureus* carriers of virulence genes isolated at the heart of nasal carriage in patients had reported a rate of 14.5% for the sea gene. While resistant genes offer protective covering of *Staphylococcus aureus* from external forces such as antimicrobials, virulent genes on the other hand ensure invasive-ness to the host cells. This intra-cooperation results in pathogenicity and the survival strategies of *Staphylococcus aureus* strains.

Sequence analysis of seven household genes for each MRSA strain classified the 12 MRSA isolates into 10 different sequence types (ST5710, ST2430, ST5289, ST5786, ST6942, ST6943, ST6944, ST6945, ST6946, ST6947). Among these, two isolates (SA32 and SA7) lukS-PV/lukF-PV-negative, were designated ST5710. The clonal type ST5710 was identified in MRSA isolates from food samples (eggs) in china in 2018 [15]. The presence of foodborne isolates in human infections can be explained by environmental and human contamination through the presence of open wounds. The lukS-PV/lukF-PV-positive SA31 isolate isolated in a hospital setting was identified as ST2430. This clonal type has been reported in community-acquired infections in Mozambique (2010) and in pyomyositis infections in Uganda (1995). The SA6 isolate, lukS-PV/lukF-PV-positive and isolated in a hospital setting, was assigned to ST5786. The presence of this clone in hospitals in England was reported in 2019. The lukS-PV/lukF-PV-negative SA9 isolate was isolated in community settings and assigned as ST5289. This clone has been reported in community infections in China (2018). The presence of clones ST5710, ST2430, ST5289, ST5786 may indicate intercontinental transmission, as these clones have been listed as identified in other countries [15].

Isolates SA2, SA4, SA5, SA34, SA12, SA21, and SA33 were assigned to new Isolates SA2, SA4, SA5, SA34, SA12, SA21 and SA33 were assigned to the new Sequences Types; ST6942, ST6943, ST6945, ST6946, ST6947 and ST6944, respectively. ST6942, ST6943, ST6945, ST6946, ST6947, and ST6944, respectively.

### Limitations of the study

-Nearly 25% of the strains collected were lost during transport between Burkina Faso and France.
- This project was part of a three-month mobility between (Laboratory of Applied Biochemistry and Immunology (LaBIA) in Burkina Faso) and (Laboratory of Bacteriology Hôpital Armaud de Villeneuve-CHU de Montpellier). These three months allowed us to analyze only 70 strains.

## Conclusion

The resistance profile of *Staphylococcus aureus* strains varies considerably by antibiotic family and type. The best antibacterial sensitivities were observed with aminosides, trimethoprim/sulfamethoxazole, clindamycin, fusidic acid, and linezolid. And the most important resistances were obtained with the β-lactam family. The study showed a diversity of antibiotic resistance phenotypes and a low rate of toxigenic strains.

The activity on the genetic diversity of MRSA has shown a great diversity of clones causing infections.

## Data Availability

Data for this research are available for this work and can be accessed from the corresponding author: ganamabasse@gmail.com and Godreuil Sylvain s-godreuil@chu-montpellier.fr. The sequences have been deposited on the NCBI BioProject site with the access code: https://www.ncbi.nlm.nih.gov/bioproject/1069879.
